# A deep learning‐based deconvolution framework identifies human brain cell‐type specific expression quantitative trait loci (eQTLs) and pathobiology in Alzheimer’s disease

**DOI:** 10.1002/alz.089093

**Published:** 2025-01-03

**Authors:** Lijun Dou, Jielin Xu, Yuan Hou, Feixiong Cheng

**Affiliations:** ^1^ Cleveland Clinic, Cleveland, OH USA; ^2^ Case Western Reserve University, Cleveland, OH USA

## Abstract

**Background:**

Cell‐type specific expression quantitative trait loci (eQTLs) can help dissect cellular heterogeneity in the impact of genetic variation on gene expression for Alzheimer’s disease (AD) and AD‐related dementia (ADRD). However, due to the high cost and stringent sample collection criteria, it is challenging to obtain large single‐nuclei RNA sequencing (snRNA‐seq) data with sufficient cohort size to match genotyping data to systematically identify human brain‐specific eQTLs for AD/ADRD.

**Method:**

In this study, we presented a deep learning‐based deconvolution framework on large‐scale bulk RNA sequencing (RNA‐seq) data to infer cell‐type specific eQTLs in the human brains with AD/ADRD. Specifically, we first predicted the brain cell‐type specific gene expression for the harmonized bulk RNA dataset (n = 1,092) from Religious Orders Study and Memory and Aging Project (ROS/MAP). We then incorporated the inferred cell‐type specific gene expression with matched whole genome sequencing (WGS) data from ROS/MAP to identify the brain cell‐type specific eQTLs. These cell‐type specific eQTLs were further colocalized with AD genome‐wide association study (GWAS) findings to discover potential risk genes and druggable targets for AD.

**Result:**

We identified 44,504 genome‐wide significant cell‐type specific cis‐eQTLs (window size = 1Mb, *p* < 5 × 10^‐8^) from eight brain cell types, including excitatory neurons, inhibitory neurons, microglia, oligodendrocytes, oligodendrocyte precursor cells, astrocytes, and endothelium. Approximate 2,732 eQTLs in astrocytes and 7,628 in excitatory neurons are identical to the results from a large existing snRNA‐seq data, associated with the regulation of multiple genes (eGenes, e.g., *ARL17B*, *LRRC37A2*, *ERAP2*, *PILRB*, *ZNF266*). We illustrated that the GWAS variant rs199456 (AD GWAS: *p* = 2.57 × 10^‐9^) co‐localized with its regulatory effect on *LRRC37A2*, *LRRC37A* and *ARL17B* gene expression in excitatory neurons.

**Conclusion:**

In summary, this study presented comprehensive brain cell type‐specific eQTL analysis and identified potential eQTL‐regulated likely causal genes from AD GWAS findings using a deep learning‐based deconvolution framework. It offers an opportunity to efficiently discover the effect of GWAS loci on gene expression at cell type‐specific manners using genotyping data and matched bulk RNA‐seq data instead of costly snRNA‐seq data. Functional validation of candidate eQTLs and associated genes are warranted in the future.

